# An Optimal Integral Controller for Adaptive Optics Systems

**DOI:** 10.3390/s23229186

**Published:** 2023-11-15

**Authors:** Pedro Escárate, María Coronel, Rodrigo Carvajal, Juan C. Agüero

**Affiliations:** 1 School of Electrical Engineering, Pontificia Universidad Católica de Valparaíso (PUCV), Av. Brasil 2147, Valparaíso 2362804, Chile; rodrigo.carvajal@pucv.cl; 2 Department of Electricity, Universidad Tecnológica Metropolitana (UTEM), Av. Jose Pedro Alessandri 1242, Santiago 7800002, Chile; macoronel@utem.cl; 3 Electronics Engineering Department, Universidad Técnica Federico Santa María, Av. España 1680, Valparaíso 2390123, Chile; juan.aguero@usm.cl

**Keywords:** adaptive optics, disturbances, identification, minimum variance, integral controller, Whittle’s likelihood

## Abstract

Integral controllers are commonly employed in astronomical adaptive optics. This work presents a novel tuning procedure for integral controllers in adaptive optics systems which relies on information about the measured disturbances. This tuning procedure consists of two main steps. First, it models and identifies measured disturbances as continuous-time-damped oscillators using Whittles´s likelihood and the wavefront sensor output signal. Second, it determines the integral controller gain of the adaptive optics system by minimizing the output variance. The effectiveness of this proposed method is evaluated through theoretical examples and numerical simulations conducted using the Object-Oriented Matlab Adaptive Optics toolbox. The simulation results demonstrate that this approach accurately estimates the disturbance model and can reduce the output variance. Our proposal results in improved performance and better astronomical images even in challenging atmospheric conditions. These findings significantly contribute to adaptive optics system operations in astronomical observatories and establish our procedure as a promising tool for fine-tuning integral controllers in astronomical adaptive optics systems.

## 1. Introduction

Adaptive optics (AO) is an optical technology that is widely used to compensate for wavefront disturbances caused by atmospheric turbulence and vibrations. These disturbances introduce aberrations into the incoming wavefront, resulting in blurred and distorted images. The basic principle behind AO involves the use of a deformable mirror, a wavefront sensor, and a control system [1]. The wavefront sensor measures the aberrations in the incoming wavefront caused by atmospheric turbulence and vibrations. In operation, the wavefront sensor samples the incoming wavefront and determines the aberrations introduced by the disturbances. The control system analyzes this information and calculates the necessary corrections to apply to the deformable mirror. The deformable mirror, a specially designed mirror composed of numerous small actuators that can deform its shape in response to commands from the control system, then adjusts its shape in real-time to compensate for the disturbances [1].

However, despite the progress made in AO technology, there are ongoing challenges related to modeling and mitigating disturbances in modern AO systems [2,3,4,5,6,7,8,9,10]. Accurate models that describe the dynamics of the AO system [11,12,13,14] and precise models for disturbances such as turbulence and vibrations are essential when implementing effective control techniques to mitigate these aberrations. In particular, disturbance mitigation is especially critical for the next generation of telescopes [15,16], which strive to achieve unprecedented levels of image quality and resolution.

Integral control is one of the most widely employed control techniques in AO systems [17,18,19,20,21]. Integral controllers are effective at mitigating low-frequency disturbances, and provide steady and accurate correction over time. Although disturbances in AO systems are dynamic phenomena, the potential to adapt and adjust controller parameters based on real-time measurements of these disturbances could represent a valuable asset for observatories.

This work proposes a novel tuning procedure for integral controllers in astronomical AO systems. The proposed tuning procedure utilizes wavefront sensor measurements recorded over an interval of 3 s to estimate the disturbance model using the modeling method in [22], which represents the operational conditions of the adaptive optics system. This approach aims to minimize the output variance, which directly impacts the quality of astronomical images. By reducing the output variance, the performance of the AO system can be optimized and improved image quality can be achieved. While recent efforts in advanced control techniques have been reported in the literature [9,10,23,24,25], there has been limited research focusing on the optimization and tuning of integral controllers in today’s astronomical AO systems.

Furthermore, this study evaluates the performance of the integral controller obtained using the proposed method, a manually tuned integral controller, and an integral controller tuned with classical methods such as Ziegler–Nichols [26]. The performance of these integral controllers was compared through theoretical examples and numerical simulations using the Object-Oriented Matlab Adaptive Optics (OOMAO) [27], an open-source software for adaptive optics simulation [27].

The rest of this paper is organized as follows. In Section 2, we provide a comprehensive overview of the AO system, introducing the description of the AO system model, typical AO controller, disturbance model, and frequency domain identification method for the disturbances. Section 3 presents the proposed method for tuning the integral controller parameter. Section 4 illustrates the controller’s performance through a simple numerical example and evaluates the proposed tuning method in an AO system using OOMAO [27]. Finally, our conclusions are presented in Section 5.

## 2. Adaptive Optics Systems

### 2.1. AO System Model

**Figure 1:**
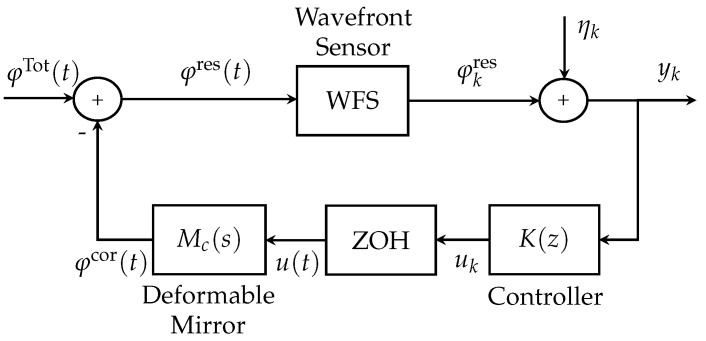
Block diagram for an AO closed-loop system.

The primary objective of the AO system is to enhance the quality of astronomical images by mitigating disturbances in the incoming wavefront. The diagram of an AO system is illustrated in Figure 1. In this figure, the incoming (aberrated) wavefront is captured by a Shark–Hartman wavefront sensor (SH-WFS) and this signal is subsequently input to the controller. Finally, the controller output represents the actuation applied to the deformable mirror (DM) to mitigate the disturbances.

In Figure 1, the signal $y_{k}$ corresponds to the output of the AO system, the signal $u_{k}$ represents the output of the controller K(z), $D_{c}\left(s\right)$ represents the continuous-time transfer function of the WFS, and $M_{c}\left(s\right)$ represents the continuous-time transfer function of the DM. The signal $\varphi^{\mathrm{Tot}}\left(t\right)$ corresponds to the total disturbance, which represents the amplitude of the disturbed wavefront caused by a combination of turbulence and vibrations. The signal $\varphi^{\mathrm{cor}}\left(t\right)$ represents the correction provided by the DM, the signal $\varphi^{\mathrm{res}}\left(t\right)$ represents the residual disturbance, and the signal $\eta_{k}$ represent the measurement noise.

The total disturbed wavefront $\varphi^{\mathrm{Tot}}\left(t\right)$ in Figure 1 is defined following [14,28,29] as the summation of the atmospheric turbulence effect $\varphi^{\mathrm{tur}}\left(t\right)$ and the effects originating from *m* different vibration sources $\varphi^{{\mathrm{vib}}_{i}}\left(t\right)$ with i=1,...,m, as follows:

(1) $$\varphi^{\mathrm{Tot}}\left(t\right)=\varphi^{\mathrm{tur}}\left(t\right)+\varphi^{{\mathrm{vib}}_{1}}\left(t\right)+\varphi^{{\mathrm{vib}}_{2}}\left(t\right)+\cdots +\varphi^{{\mathrm{vib}}_{m}}\left(t\right).$$

Discrete-time additive white Gaussian noise with a mean of zero is part of the wavefront sensor output signal model (see Figure 1, where it is denoted by $\eta_{k}$). For the simplicity of presentation, the atmospheric turbulence $\varphi^{\mathrm{tur}}$, vibrations $\varphi^{\mathrm{vib}}$, and measurement noise η are all assumed to be mutually independent.

It is important to note that both discrete-time and continuous-time signals are present in Figure 1. The input of the WFS is a continuous-time signal, whereas its output is a discrete-time signal. Additionally, AO systems incorporate a zero-order hold (ZOH) [14,29,30] to convert the discrete-time control signal into a continuous-time control signal applied to the deformable mirror.

The WFS plays a crucial role in estimating the residual phase by accumulating photons over a time interval Δ and integrating the residual phase $\varphi^{\mathrm{res}}\left(t\right)$ within the sampling time interval $\left[t_{k-1},t_{k}\right]$, where $t_{k}=k\Delta$ denotes the sampling instant [14]. Consequently, the discrete-time residual phase can be mathematically expressed as follows:

(2) $$\varphi_{k}^{\mathrm{res}}=\frac{1}{\Delta }\int_{t_{k-1}}^{t_{k}}\varphi^{\mathrm{res}}\left(t\right)dt,$$

where $k\in N_{0}$ and Δ is the sampling period. Typically, the WFS in (2) is modeled as the following discrete-time transfer function [14]:

(3) $$D\left(z\right)=\frac{D_{0}}{z^{\mu }},$$

where $D_{0}$ is the gain and μ is the measurement delay.

On the other hand, the DM is modeled as the following discrete-time transfer function [11,14,28,31,32]:

(4) $$M\left(z\right)=\frac{M_{0}}{z^{\tau }},$$

where $M_{0}$ is the gain and τ is the correction delay.

Finally, improved image quality can be achieved by minimizing either the continuous-time phase variance, the output of the AO system $\mathrm{var}\left\{y_{k}\right\}$, or the variance of residual phase (denoted as $\mathrm{var}\left\{\varphi^{\mathrm{res}}\left(t\right)\right\}$), as described in [14], where the authors demonstrated that minimizing the discrete-time residual phase variance $\mathrm{var}\left\{\varphi_{k}^{\mathrm{res}}\right\}$ is equivalent to minimizing the continuous-time residual phase variance (see, e.g., [14,31]).

### 2.2. AO Controller

As previously mentioned, integral control is widely employed as a control algorithm in AO systems [17,18,19,20,21]. The control law for an integral controller is defined as follows [33,34]:

(5) $$u_{k}=u_{k-1}-K_{I}y_{k},$$

which results in the following transfer function:

(6) $$K\left(z\right)=\frac{K_{I}}{z-1}.$$

In order to address the complexity of the control problem, a commonly employed approach is to represent disturbances as a linear combination of orthogonal basis functions known as “modes”. In this work, we utilize Zernike polynomials [35], which are common orthogonal basis functions for describing optical aberrations. This approach allows the original control problem to be separated into independent modes. In this study, we simulated the first 100 Zernike modes. However, to effectively demonstrate the proposed tuning method, we focused solely on the first three modes, called Tip, Tilt, and Defocus [1,36], as these modes are particularly sensitive to vibrations [1,2,31]. The piston mode was not considered, as it cannot be measured in practical applications by the WFS used in this work.

Despite the widespread use of integral controllers in AO, determining the optimal value of $K_{I}$ based on varying disturbance conditions remains challenging due to the time-varying nature of perturbations. This work presents a novel method for tuning an integral controller to tackle this challenge. In the proposed approach, the control parameter is determined based on the disturbance model and the plant model. This method can enable real-time tuning of the controller to adapt to specific disturbances, leading to improved performance and enhanced correction accuracy. Moreover, this technique is expected to contribute to the advancement of more sophisticated AO systems, ultimately facilitating the acquisition of improved astronomical images.

### 2.3. Disturbance Model

In AO systems, disturbances such as turbulence and vibrations are typically described using discrete-time second-order autoregressive (AR) models, as shown in [17,29,37]. However, in this study we consider an alternative approach utilizing a sum of continuous-time oscillators, as proposed in [22]. Consequently, the continuous-time model for these disturbances is described as follows:

(7) $$\varphi^{\mathrm{Tot}}\left(t\right)=\sum_{l=1}^{\rho }\frac{\gamma_{l}}{s^{2}+2\psi_{l}\varpi_{l}s+\varpi_{l}^{2}}{\dot{\nu }}_{l}\left(t\right),$$

where $s=\frac{d}{dt}$ is the derivative operator, ρ represents the number of oscillators, $\gamma_{l}$ is the gain, $\varpi_{l}$ (in rads/s) is the natural frequency $\left(\varpi_{l}=2\pi \chi_{l}\right)$, $\psi_{l}$ is the damping coefficient, and ${\dot{\nu }}_{l}\left(t\right)$ is continuous-time zero-mean white Gaussian noise with variance $\sigma_{l}^{2}=1\delta_{D}\left(t\right),\forall l$ (with $\delta_{D}\left(t\right)$ being the Dirac delta). In addition, ${\dot{\nu }}_{l}\left(t\right)$ and l=1⋯,ρ are assumed to be mutually uncorrelated noise.

**Figure 2:**
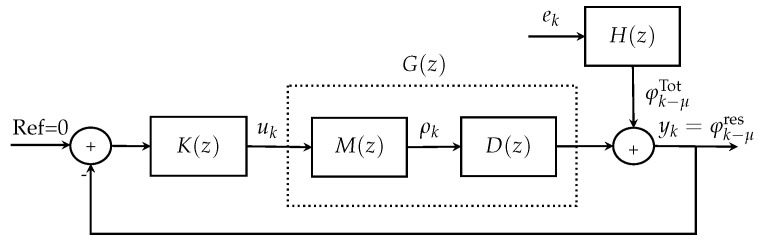
Equivalent block diagram model for AO system.

Figure 2 represents an equivalent block diagram model for the AO system presented in Figure 1. As presented in [22], the AO disturbances in discrete time can be obtained by solving a Riccati equation [26,33,38] to compute the spectral factorization H(z) [33] via numerical approximation. Thus, the discrete-time model of the disturbances is as follows (for more details, see Appendix B and [22]):

(8) $$\varphi_{k}^{\mathrm{Tot}}=H\left(z\right)e_{k},$$

where

(9) $$H\left(z\right)=\sum_{l=1}^{\rho }\frac{z^{2}+c_{1l}z+c_{2l}}{z^{2}+d_{1l}z+d_{2l}}$$

and $e_{k}$ is discrete-time zero-mean white Gaussian noise with variance $\varsigma^{2}$. It is essential to remark that both H(z) and $\varsigma^{2}$ depend on the continuous-time parameters.

In Figure 2, it can be observed that the plant is G(z)=M(z)D(z). Here, it is assumed that the plant is known and that the disturbances are identified using the identification method presented in [22].

### 2.4. Identification in Frequency Domain

In this work, in order to facilitate the implementation of the proposed tuning method in real-time AO operations while achieving a precise estimation of the disturbance model parameters, we carry out the identification of the disturbance parameters in the frequency-domain using Whittle’s likelihood [39,40,41]. The choice of this identification method is based on the equivalence between time-domain and frequency-domain maximum likelihood identification methods, as demonstrated in [42]. The proposed identification method used in this work requires us to estimate the vector of the parameters, denoted as $\vec{\theta }$ and defined as follows:

(10) $$\vec{\theta }={\left[\begin{array}{ccc} {\vec{\chi }}^{T} & {\vec{\psi }}^{T} & {\vec{\gamma }}^{T} \end{array}\right]}^{T},$$

where $\vec{\chi }={\left[\chi_{1}\;\;\;\chi_{2}\;\;\;\cdots \;\;\;\chi_{\rho }\right]}^{T}$, $\vec{\psi }={\left[\psi_{1}\;\;\;\psi_{2}\;\;\;\cdots \;\;\;\psi_{\rho }\right]}^{T}$, and $\vec{\gamma }={\left[\gamma_{1}\;\;\;\gamma_{2}\;\;\;\cdots \;\;\;\gamma_{\rho }\right]}^{T}$ are the natural frequencies (in Hz), damping coefficients, and gains, respectively, of the disturbance model in (7).

The Whittle likelihood function depends on the discrete-time Power Spectral Density (PSD) of the disturbances $\Phi (e^{i\omega_{j}\Delta })$ and the periodogram of the sampled-data series of the measured $\varphi^{\mathrm{Tot}}$, $I(e^{i\omega_{j}\Delta })$. This likelihood function for sampled data and finite data length was presented in [22], and is provided by

(11) $$l_{N}\left(\vec{\theta }\right)=-\frac{1}{N}\sum_{j=1}^{N}\log\Phi \left(e^{i\omega_{j}\Delta }\right)-\frac{1}{N}\sum_{j=1}^{N}\frac{\Delta I(e^{i\omega_{j}\Delta })}{\Phi (e^{i\omega_{j}\Delta })},$$

where *N* is the data length, Δ is the sampling period, $\Phi (e^{i\omega_{j}\Delta })$ is given by:

(12) $$\Phi \left(e^{i\omega_{j}\Delta }\right)={\left|H(e^{i\omega_{j}\Delta })\right|}^{2}\varsigma^{2},$$

$i=\sqrt{-1}$, j=1,2,⋯,N, $\omega_{j}=\left(2\pi j\right)/\Delta$, $0<\omega_{j}\leq 2\pi \left(F_{s}/2\right)$, and $I(e^{i\omega_{j}\Delta })$ in (11) is obtained from the discrete Fourier transform (DFT) of the data as provided by [22]

(13) $$I\left(e^{i\omega_{j}\Delta }\right)={\left|\frac{1}{\sqrt{N}}\sum_{t=1}^{N}\varphi_{t}^{\mathrm{Tot}}e^{-i\omega_{j}\Delta }\right|}^{2}.$$

The maximum likelihood (ML) estimation problem can be formulated as follows [22]:

(14) $${\hat{\theta }}_{\mathrm{ML}}=\arg\min_{\vec{\theta }}\;-l_{N}\left(\vec{\theta }\right),\;\;s.t.\;\;\left\{\begin{array}{c} 0<\chi_{l}<F_{s}/2\\ 0<\psi_{l}<1\\ \gamma_{l}>0 \end{array}\right.,\;l=0,\cdots ,\rho .$$

Finally, solving the minimization problem yields a disturbance model adjusted to the disturbances measured by the WFS. It is important to note that for the successful application of this method it is necessary to know the total disturbance ($\varphi^{\mathrm{Tot}}$). In a real AO system, this task can be accomplished in open-loop mode using a flat DM. Consequently, the novel approach presented in this work can serve as a valuable tool for real-time AO operations, enabling adjustment of the disturbance model based on a set of N samples of the total disturbance ($\varphi^{\mathrm{Tot}}$) measured by the WFS and subsequent fine-tuning of the integral controller to enhance the performance of the AO system.

## 3. Proposed Method

To fine-tune the integral controller, the proposed method deals with determining the optimal gain for the integral controller, denoted as $K_{I}$, that minimizes the variance of the output signal $\mathrm{var}\left\{y_{k}\right\}$ [43]. This approach represents a viable strategy for enhancing image quality. According to the block diagram in Figure 2, the output signal is defined as follows:

(15) $$y_{k}=G\left(z\right)u_{k}+H\left(z\right)e_{k},$$

where (in an abuse of notation) *z* is either the forward shift operator $\left(zy_{k}=y_{k+1}\right)$ or the argument of the Z-Transform, G(z) is the discrete-time transfer function of the plant, which is provided by

(16) $$G\left(z\right)=\frac{B(z)}{A(z)},$$

with

(17) $$A\left(z\right)=z^{n}+a_{1}z^{n-1}+\cdots +a_{n-1}z+a_{n},$$

(18) $$B\left(z\right)=b_{0}z^{m}+b_{1}z^{m-1}+\cdots +b_{m-1}z+b_{m},$$

$u_{k}$ is the output of the controller as provided by (5), H(z) is the discrete-time transfer function of the disturbances, and the signal $e_{k}$ is zero-mean white noise with variance $\varsigma^{2}$ (see Equation (8)). Observe that the relative order of G(z) is d=n−m, which can be considered as the delay of the system in terms of the backward-shift operator.

In order to obtain the integration constant $\left(K_{I}\right)$ for an integral controller, it is considered that the closed-loop response is provided by

(19) $$y_{k}=H\left(z\right)S\left(z\right)e_{k},$$

where H(z) and $e_{k}$ represent the disturbance model in (15) and S(z) is the sensitivity function provided by

(20) $$S\left(z\right)=\frac{1}{1+G(z)K(z)},$$

where K(z) is the controller. We assume that the structure of the integral controller for min-phase systems is the Z-transform of the control law in (5); it is provided by

(21) $$K\left(z\right)=\frac{K_{I}z}{z-1},$$

where the $K_{I}$ is the integration constant.

Next, to minimize the output signal variance, we seek to minimize the following cost function:

(22) $$V=\varsigma^{2}{∥H(z)S(z)∥}_{2}^{2},$$

through replacement, we obtain   

(23) $$\begin{array}{cc} V & =\varsigma^{2}{∥\frac{H(z)}{1+G(z)K(z)}∥}_{2}^{2},\\ \; & =\varsigma^{2}{∥\frac{H(z)A(z)(z-1)}{A\left(z\right)\left(z-1\right)+B\left(z\right)K_{I}z}∥}_{2}^{2}, \end{array}$$

where H(z) and $\varsigma^{2}$ represent the model of disturbance in (15), B(z) and A(z) represent the plant, and $K_{i}$ is the integration constant.

To assure stability, it must be guaranteed that the polynomial $A\left(z\right)\left(z-1\right)+B\left(z\right)K_{I}z$ has all of its roots inside the unit circle of the *z*-plane, using, e.g., the Jury stability test [44]. Lemma A1 shows an example of conditions for the stability of a second-order polynomial. Note that H(z) is not considered in the stability analysis due to its being obtained from the spectral factorization procedure, which is a stable result (for more information, see [22]).

It is important to note that in this case it is not necessary to include the measurement noise model to obtain $K_{I}$, as this noise is white and independent of the disturbances. Due to this, the resulting controller gain remains the same with or without the term associated with the measurement noise.

**Remark 1.**  *Note that in our modeling approach, all of the perturbations are encompassed in a single transfer function H(z) via spectral factorization. This implies that the solution to the problem of obtaining a controller that minimizes the variance of the output of the control loop is provided by the inherently stabilizing Minimum Variance Controller (MVC); see, e.g., [31]. However, unlike our problem of interest, MVC does not enforce any given structure for the controller. This implies that when we have a desired structure for the controller, the resulting optimization problem is typically not convex, making it challenging to solve. In our problem of interest, we only have one variable to optimize, potentially yielding a simpler optimization problem.*

## 4. Results and Discussion

In this section, we illustrate the benefits of our proposal through two examples. First, we analyze the effectiveness of our tuning method on a simple second-order system with second-order disturbances. Second, we analyze an AO system where the disturbances are modeled as an *n*th-order system.

### 4.1. Tuning Controller Example

In this example, the plant’s transfer function and the disturbances model are assumed to be known. The transfer functions for the plant and disturbances are as follows:

(24) $$G\left(z\right)=\frac{3z+1.5}{z(z^{2}-1.5z+0.7)},$$

(25) $$H\left(z\right)=\frac{50z-20}{z^{2}-0.2z+0.75}.$$

We utilize white noise with a variance of $\varsigma^{2}=1$, a sampling period of Δ=1 ms, and a data length of N=1000 samples. To assess the performance of the proposed method, we consider an alternative tuning approach for comparison, namely, the classical Ziegler–Nichols method [45].

**Figure 3:**
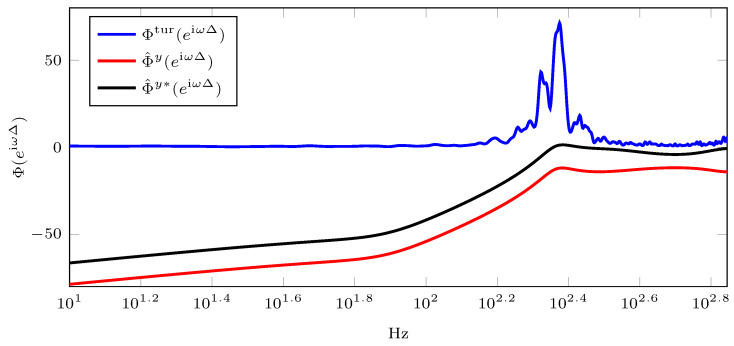
Discrete-time PSD of the disturbance (input signal) and controlled outputs (with integral controller). The blue line represents the discrete-time PSD of disturbance or input signal, the red line represents the discrete-time PSD of the controlled output using the proposed tuning method, and the black line represents the discrete-time PSD of the controlled output using the Ziegler–Nichols tuning method.

Figure 3 shows the discrete-time PSD of the controlled output. The red line (${\hat{\Phi }}^{y}\left(e^{i\omega \Delta }\right)$) represents the proposed tuning, while the black line (${\hat{\Phi }}^{y*}\left(e^{i\omega \Delta }\right)$) represents the Ziegler–Nichols tuning. It should be noted that these controlled outputs are obtained in a closed-loop configuration. Additionally, the blue line ($\Phi^{\mathrm{tur}}\left(e^{i\omega \Delta }\right)$) represents the discrete-time PSD of the disturbance signal in the open loop, corresponding to the input signal. As shown in Table 1, it can be observed that the controlled output using the proposed tuning method exhibits better performance in terms of the output variance.

The simulation time was arbitrarily chosen as N=1000 to ensure enough samples for estimating the disturbance model and simulating the system. It is important to note that this example presents a system in which the transfer function of the system G(z) and transfer function of the disturbances H(z) are both known. Therefore, the proposed method can be applied to tune an integral controller for any system where G(z) and H(z) are known or estimated.

### 4.2. Tuning a Controller for an Adaptive Optics System

In this example, we present simulations to assess the performance of the integral controller tuning procedure in an AO system, as detailed in the preceding sections. These simulations were conducted using the Object-Oriented Matlab Adaptive Optics Toolbox software [27]. An overview of the simulation parameters utilized in this study is presented in Table 2.

The chosen values of the Fried parameter $r_{0}$ used in the simulations correspond to two scenarios: one representing typical atmospheric conditions for astronomical observations ($r_{0}=14$) and the other representing favorable atmospheric conditions for astronomical observations ($r_{0}=20$) [46,47,48]. This deliberate selection enabled us to evaluate the performance of the proposed method under varying disturbance conditions.

**Figure 4:**
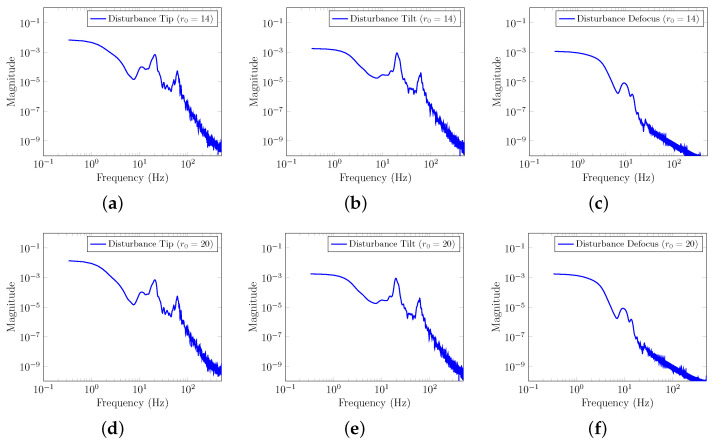
Discrete-time PSD of the total disturbance signal. (**a**) Tip mode PSD for $r_{0}=14$. (**b**) Tilt mode PSD for $r_{0}=14$. (**c**) Defocus mode PSD for $r_{0}=14$. (**d**) Tip mode PSD for $r_{0}=20$. (**e**) Tilt mode PSD for $r_{0}=20$. (**f**) Defocus mode PSD for $r_{0}=20$.

As described in Section 2, the total disturbance was decomposed into different Zernike modes. Figure 4 illustrates the discrete-time Power Spectral Density (PSD) of the total disturbance for the first three Zernike modes (Tip, Tilt, Defocus). We excluded the Piston mode from our analysis because the SH-WFS cannot measure it. It is worth noting that vibration peaks at 20 Hz and 60 Hz have been introduced into the Tip and Tilt Zernike modes, similar to those considered in [22], to account for vibrations commonly encountered in AO systems [6].

To successfully implement the proposed method, as outlined in Section 3, it is necessary to have models for both the plant and the disturbances. In this work, we utilized a commonly employed AO plant model [14,22,49], represented as follows:

(26) $$G\left(z\right)=\frac{D_{0}M_{0}}{z^{d}},$$

The model parameters were set to $D_{0}=M_{0}=1$ with d=1, consistent with the values used in [22]. The method outlined in Section 2.4 and Algorithm A1 (presented in Appendix B) were used to identify the disturbance model, with nine oscillators (ρ=9) used to model the Tip, Tilt, and Defocus Zernike modes.

**Figure 5:**
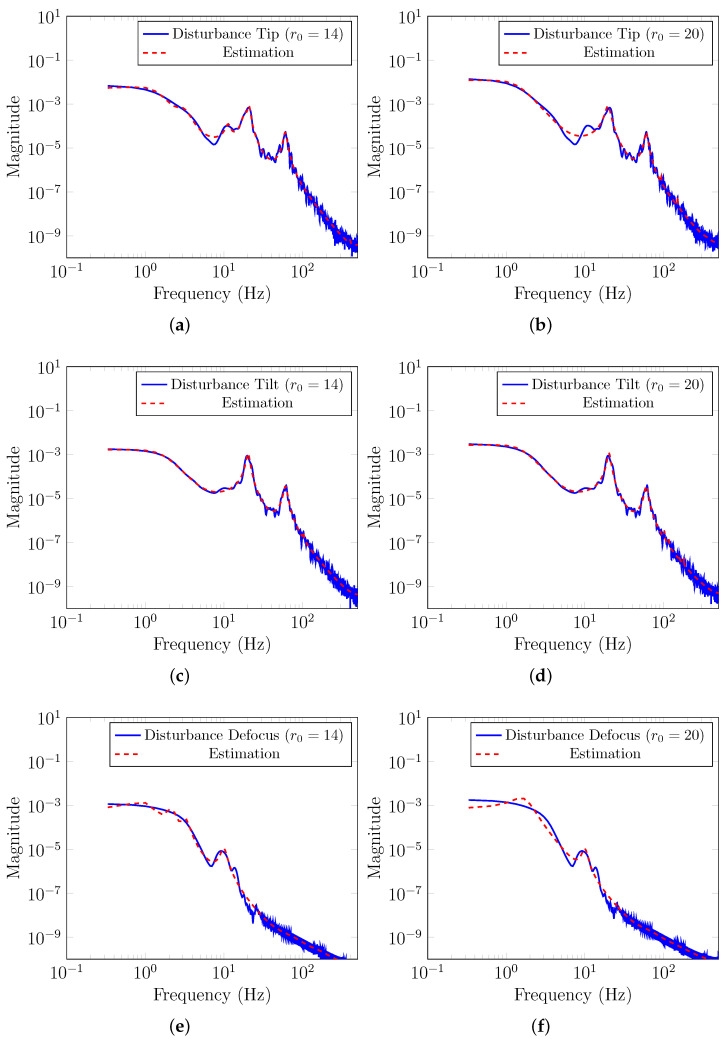
Estimated PSD for Zernike modes. (**a**) Tip mode PSD estimation for $r_{0}=14$. (**b**) Tip mode PSD estimation for $r_{0}=20$. (**c**) Tilt mode PSD estimation for $r_{0}=14$. (**d**) Tilt mode PSD estimation for $r_{0}=20$. (**e**) Defocus mode PSD estimation for $r_{0}=14$. (**f**) Defocus mode PSD estimation for $r_{0}=20$.

Figure 5 illustrates the estimation results obtained using the identification method proposed in [22] for the first three Zernike modes. In this figure, the blue line represents the discrete-time Power Spectral Density (PSD) of the total disturbance generated with OOMAO, while the red line represents the PSD of the estimated disturbance model using the identification method proposed in [22]. Clearly, the identification method yields high accuracy for the Tip, Tilt, and Defocus Zernike modes. Notice that for the Tip and Tilt modes we were able to successfully and accurately detect model the vibration peaks at 20 Hz and 60 Hz.

With the disturbance model estimated, the integral controller gain $K_{I}$ can be determined by solving the estimation problem described in Equation (23) using the optimization function fmincon in Matlab Version 9.12.0 (R2022b) [50].

To compare and evaluate the performance of the proposed method in an adaptive optics system, we proposed a simulation study using three different tuning methods: (a) manual tuning, (b) the Ziegler–Nichols tuning method, and (c) tuning using the proposed method. An integral controller was tuned by each method and compared in terms of the Root Mean Square (RMS) of the residual disturbance variance, with the superior controller being the one that obtains the lowest RMS value.

In this work, the RMS value of the residual disturbance measured using OOMAO [27] was obtained as follows:

(27) $$RMS=\sqrt{\mathrm{var}\left\{\varphi_{k}^{\mathrm{res}}\right\}}\frac{\lambda }{2\pi }.$$

With manual tuning, values of $K_{I}$ between 0.045 and 0.050 were evaluated to tune an integral controller. The best outcome was achieved with $K_{I}=0.045$ for $r_{0}=14$ and $K_{I}=0.050$ for $r_{0}=20$. On the other hand, because the Ziegler–Nichols method depends solely on the transfer function G(z), a value of $K_{I}$ = 0.0203 was determined for Tip, Tilt, and Defocus modes in both scenarios for $r_{0}=14$ and $r_{0}=20$. Finally, in case (c), to determine the values of $K_{I}$, we minimized the cost function from (23) in terms of $K_{I}$, then used Appendix A to consider the stability restriction, obtaining the following results: $K_{I}=0.0367$ for Tip mode, $K_{I}=0.0212$ for Tilt mode, and $K_{I}=0.1232$ for Defocus mode with $r_{0}=14$; and with $r_{0}=20$, $K_{I}=0.0384$ for Tip mode, $K_{I}=0.0222$ for Tilt mode, and $K_{I}=0.1288$ for Defocus mode.

The mean values of the obtained residual disturbance variance RMS are summarized in Table 3. The results indicate that the integral controller tuned using the proposed method outperforms the manually tuned integral controller and the Ziegler–Nichols tuned integral controller. It is clear that the proposed method provides better tuning of the integral controller. Even though the manual and Ziegler–Nichols tuning both exhibit good performance, the integral controller tuned using the proposed method achieves superior results, outperforming the manual tuning method in terms of RMS residual turbulence by 7.72% for $r_{0}=14$ and by 6.39% for $r_{0}=20$. Because the proposed method requires a window of N=1000 samples to estimate the disturbance model and calculate the integral controller gain $K_{I}$, it is suitable for implementation in astronomical real-time AO systems, resulting in a time window of approximately 1 s in current AO systems. Finally, the proposed tuning method could be a valuable tool to enhance the image quality of existing AO systems using integral controllers. However, due to the dynamic nature of atmospheric conditions during nighttime observations, it is advisable to regularly adjust the integral controller, especially when changing the scientific target.

**Figure 6:**
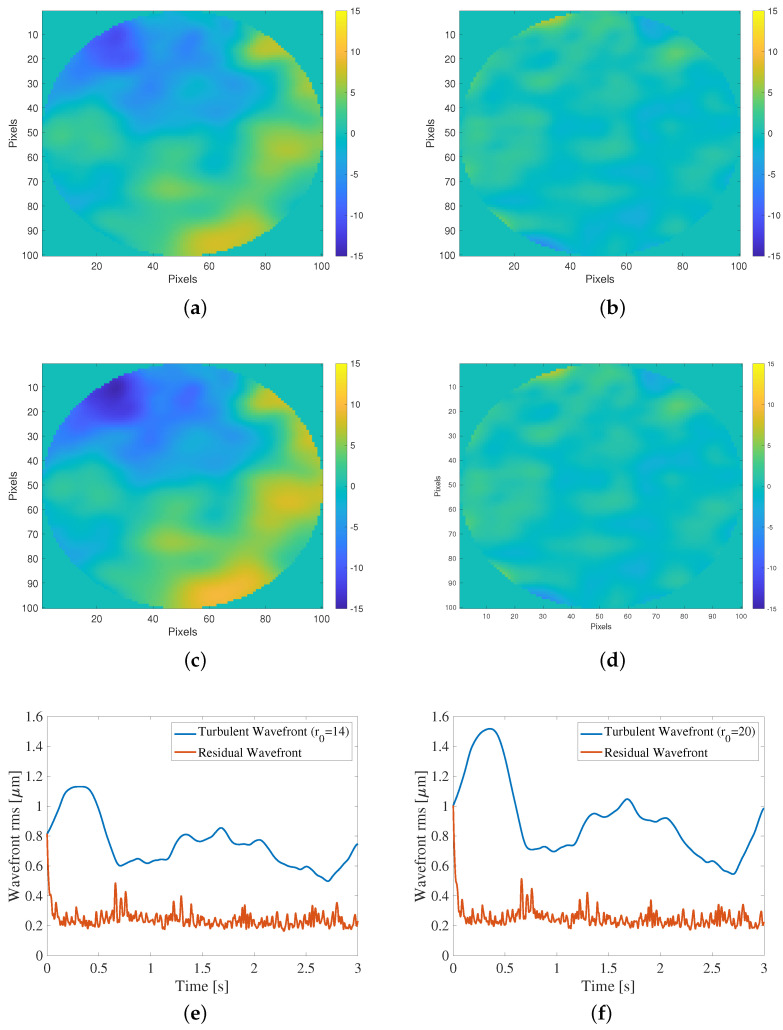
Performance of the integral controller tuned with the proposed method. (**a**) Total disturbance $\varphi^{\mathrm{Tot}}\left(t\right)$ at the end of the simulation for $r_{0}=14$. (**b**) Residual disturbance $\varphi^{\mathrm{res}}\left(t\right)$ for $r_{0}=14$. (**c**) Total disturbance $\varphi^{\mathrm{Tot}}\left(t\right)$ at the end of the simulation for $r_{0}=20$. (**d**) Residual disturbance $\varphi^{\mathrm{res}}\left(t\right)$ for $r_{0}=20$. (**e**) The blue line represents the total disturbance variance, while the red line represents the residual disturbance variance for $r_{0}=14$. (**f**) The blue line represents the total disturbance variance, while the red line represents the residual disturbance variance for $r_{0}=20$.

Further details of the control performance using our proposal are shown in Figure 6. Here, the total disturbance $\varphi^{\mathrm{Tot}}\left(t\right)$ and residual disturbance $\varphi^{\mathrm{res}}\left(t\right)$ at the end of the simulation for the two atmospheric conditions ($r_{0}=14$ and $r_{0}=20$) are illustrated. Figure 6a,b shows the total disturbance $\varphi^{\mathrm{Tot}}\left(t\right)$ for $r_{0}=14$, Figure 6c,d illustrates the residual disturbance $\varphi^{\mathrm{res}}\left(t\right)$ for $r_{0}=20$, and Figure 6e,f presents the evolution of the total disturbance and residual disturbance during the simulation for the two scenarios with $r_{0}=14$ and $r_{0}=20$. The results depicted in Figure 6 shows excellent performance in both scenarios on the part of the integral controller proposed here, which can be explained by the fact that the output variance is minimized to optimize the controller. Figure 6b,d shows that the residual disturbance is close to zero, which is expected when the controller is operating correctly. Additionally, Figure 6e,f demonstrates that the integral controller effectively reduces the output variance and enhances image quality, as expected considering its common use in AO systems.

## 5. Conclusions

This paper introduces a novel procedure for tuning integral controllers in AO systems. The proposed method utilizes the approach outlined in [22] to accurately estimate the discrete-time disturbance model using OOMAO data. By formulating a minimization problem, the method determines the optimal gain for the integral controller that minimizes the output variance. This study encompasses theoretical and numerical examples while considering unfavorable atmospheric scenarios commonly encountered in astronomical observations. Our results demonstrate the effectiveness of the proposed approach in accurately estimating disturbance models in simulated AO systems using OOMAO software. Notably, the procedure successfully minimizes the output variance, achieving a 7.73% reduction in terms of residual variance RMS for $r_{0}=14$ and a 6.39% reduction for $r_{0}=20$. These improvements can enhance the quality of astronomical images even under challenging atmospheric conditions. These findings constitute a significant contribution to the field of AO operations in astronomical observatories, establishing our procedure as a promising tool for tuning integral controllers in astronomical AO systems and enabling optimization based on specific observational conditions. Additionally, although the proposed method was evaluated for an AO system, it can be applied to any system for which the transfer functions and disturbances of the system are known.   

## Figures and Tables

**Table 1:** Performance of integral controller.

	Proposed Tuning	Ziegler-Nichols Tuning
Variance	30.18	411.91

**Table 2:** OOMAO parameters summary for AO simulations.

Parameter	Value	Unit
Diameter D	8.4	m
Shack-Hartmann WFS	10 × 10	Lenslets
Deformable Mirror	11 × 11	Number Actuators
Zernike Modes	100	-
Wavelenght λ	550	nm
Fried $r_{0}$	14–20	cm
Windspeed	15	m/s
Outer scale $L_{0}$	20	m
Sampling Time	0.001	s
Simulation Time	3	s

**Table 3:** Residual disturbance variance in RMS.

Fried Parameter	Manual Tuning	Ziegler-Nichols Tuning	Proposed Tuning
$r_{0}=14$	0.2628	0.3245	0.2426
$r_{0}=20$	0.2708	0.3484	0.2535

## Data Availability

The article provides all the information required to conduct the simulations. Data sharing is not applicable to this article.

## Abbreviations

    The following abbreviations are used in this manuscript:

AO	Adaptive Optics
OOMAO	Object-Oriented Matlab Adaptive Optics
SH	Shark–Hartman
WFS	Wavefront Sensor
DM	Deformable Mirror
PSD	Power Spectral Density
DFT	Discrete Fourier Transform
FIR	Finite Impulse Response
ML	Maximum Likelihood
PI	Proportional Integral
ZOH	Zero-Order Hold
MVC	Minimum Variance Controller
RMS	Root Mean Square

## Appendix A. Lemma

**Lemma A1.** 
*Set a discrete time second-order polynomial:*

(A1)
$$A\left(z\right)=z^{2}+a_{1}z+a_{0}.$$

*The necessary and sufficient conditions for the polynomial A(z) to have no roots on or outside the unit circle of the z-plane are:   *

(A2)
$$\begin{array}{cc} |a_{0}| & <1, \end{array}$$

(A3)
$$\begin{array}{cc} |a_{1}| & <1+a_{0}. \end{array}$$

**Proof.** See [44].    □

## Appendix B. Algorithm

The procedure to obtain the discrete-time disturbance model in Algorithm A1 is summarized in the following (for more detail, see [22]).

**Algorithm A1** Discrete-time disturbance model
1: Obtain the continuous-time state-space model of the disturbances using the following equations: (A4) $$\dot{x}\left(t\right)=A_{c}x\left(t\right)+\kappa_{c}\Psi_{c}\left(t\right),$$ (A5) $$\varphi^{\mathrm{Tot}}\left(t\right)=\dot{b}\left(t\right)=C_{c}x\left(t\right),$$ where $A_{c}\in R^{2\rho \times 2\rho }$, $x\left(t\right)\in R^{2\rho \times 1}$, $\kappa_{c}\in R^{2\rho \times \rho }$, $\Psi_{c}\in R^{\rho \times 1}$, $C_{c}\in R^{1\times 2\rho }$, ρ is the number of damping oscillators used, and the noise variance is $Q_{c}=\kappa_{c}\kappa_{c}{}^{T}$. 2: Obtain the continuous-time state-space model of the extended system: (A6) $$\dot{X}\left(t\right)=AX\left(t\right)+\left[\begin{array}{c} \kappa_{c}\\ 0 \end{array}\right]\Psi_{c}\left(t\right),$$ where (A7) $$A=\left[\begin{array}{cc} A_{c} & 0\\ C_{c} & 0 \end{array}\right].$$ 3: Compute the discrete-time state equation of the extended system: (A8) $$X_{k+1}=e^{A\Delta }X_{k}+n_{k+1}.$$ 4: Compute the discrete-time system matrices, *A* and *C*, of the disturbances using the following equations: (A9) $$A=e^{A_{c}\Delta },$$ (A10) $$C=C_{c}A_{c}{}^{-1}\left\{A-I\right\}.$$ 5: Obtain the spectral factorization H(z) and $\varsigma^{2}$.
